# Early Autumn Senescence in Red Maple (*Acer rubrum* L.) Is Associated with High Leaf Anthocyanin Content

**DOI:** 10.3390/plants4030505

**Published:** 2015-08-05

**Authors:** Rachel Anderson, Peter Ryser

**Affiliations:** Department of Biology, Laurentian University, 935 Ramsey Lake Road, Sudbury, ON P3E 2H6, Canada; E-Mail: raymaryca@yahoo.ca

**Keywords:** *Acer rubrum*, anthocyanins, autumn senescence, time of senescence, SLA, leaf dry matter content, tannins

## Abstract

Several theories exist about the role of anthocyanins in senescing leaves. To elucidate factors contributing to variation in autumn leaf anthocyanin contents among individual trees, we analysed anthocyanins and other leaf traits in 27 individuals of red maple (*Acer rubrum* L.) over two growing seasons in the context of timing of leaf senescence. Red maple usually turns bright red in the autumn, but there is considerable variation among the trees. Leaf autumn anthocyanin contents were consistent between the two years of investigation. Autumn anthocyanin content strongly correlated with degree of chlorophyll degradation mid to late September, early senescing leaves having the highest concentrations of anthocyanins. It also correlated positively with leaf summer chlorophyll content and dry matter content, and negatively with specific leaf area. Time of leaf senescence and anthocyanin contents correlated with soil pH and with canopy openness. We conclude that the importance of anthocyanins in protection of leaf processes during senescence depends on the time of senescence. Rather than prolonging the growing season by enabling a delayed senescence, autumn anthocyanins in red maple in Ontario are important when senescence happens early, possibly due to the higher irradiance and greater danger of oxidative damage early in the season.

## 1. Introduction

Development of the bright colours of many deciduous trees and shrubs during autumn senescence is arguably the most spectacular and attractive form of plant senescence. While the yellows and oranges are a result of unmasking the carotenoids during chlorophyll degradation, and the browns oxidation products of phenolics associated with cell death, the reds are mostly caused by *de novo* synthesis of anthocyanins [1]. The reasons for a plant to invest in the biosynthesis of new compounds in senescing leaves soon to be lost have been questioned for a long time [2] and still are a topic of an intense debate [3]. Theories about the role of anthocyanins range from an extravagancy without a vital function [1], excretions to detoxify the plant [4], a warning signal to deter herbivores [5,6] to an important factor for protecting leaves against excessive light [7]. Nevertheless, during the last few decades considerable evidence has been accumulating to indicate the importance of anthocyanins as photoprotectants in senescing leaves [8,9,10]. Leaves with anthocyanin tolerate more light than green leaves before photosystem II is inhibited [11] and anthocyanin-deficient mutants of deciduous shrubs are more easily damaged by photo-oxidative stress than wild types with anthocyanin [12]. Photoprotection is considered especially important during senescence as the energy of photons is not effectively harnessed anymore by photosynthesis [13]. In fully functional cells, the elaborate xanthophyll system dissipates excess energy, but anthocyanins would provide a biochemically more parsimonious alternative to this in a senescing leaf [14].

Associated with the question of the functional role of anthocyanins in senescing leaves is the question about the reasons for the wide variation in the intensity of the phenomenon, including geographic, interspecific and phenotypic differences. While a large number of species from tropics to tundra develop anthocyanins in response to wounding and stress, leaf reddening during senescence is characteristic especially for species in the deciduous zones of eastern North America and eastern Asia [6], and also in the arctic [15]. Even in these regions, though, there is wide variation among species and among genotypes of a species [16,17]. Accumulation of anthocyanins during senescence also depends on environmental conditions, their development being especially pronounced under conditions of bright light and cool temperatures [2], N-limitation [18] and P-limitation [19].

**Figure 1:**
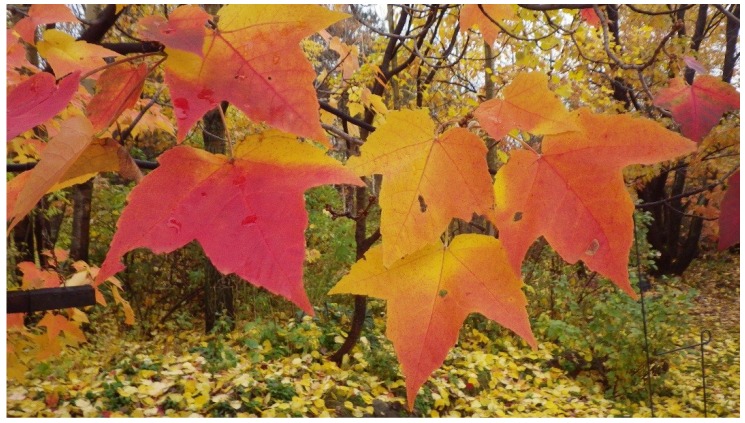
Variation in the red colour during senescence within leaves of *Acer rubrum* as a result of partial shading by other leaves. Pictures taken on 18 October 2014.

**Figure 2:**
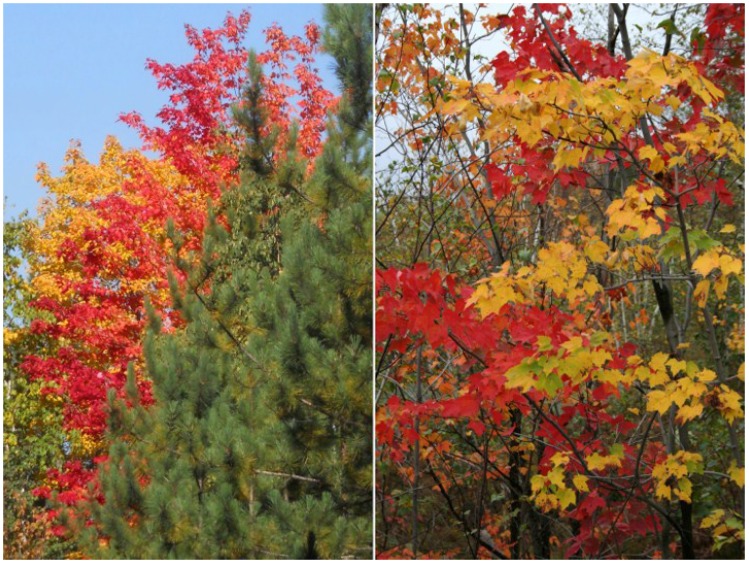
*Acer rubrum* trees vary in the degree of anthocyanin accumulation in their leaves during autumn senescence, even among trees with a similar exposure to light. Pictures taken in Sudbury, Ontario, Canada on 25 September 2014 (**left**), and 8 October 2007 (**right**).

One of the species with a large variation in the degree of redness during senescence is red maple (*Acer rubrum* L.). Red maple is a common tree in eastern North America, with a distribution from Florida to the northern limit of the deciduous biome in Ontario [20], with a high morphological and genetic variability [21], and with a wide ecological range [22]. The species is characterized by development of bright red colour during its senescence, but there is wide individual variation even in local populations, with some trees remaining yellow. Variation in the colouring is at least partly genetic, and the red autumn colour is the most pronounced in northern genotypes [16,23]. Within a tree, exposure to sunlight promotes the red colour (Figure 1), but tree-to tree variation can be observed among similarly exposed trees (Figure 2).

The goal of this project was to gain a better understanding of the inter-individual variation in the development of red colour in senescing leaves of red maple. For that purpose we studied 27 red maple trees over two growing seasons, the trees growing in close vicinity to each other, but showing large variation in their autumn colour. Assuming an adaptive advantage of anthocyanins with respect to senescence, we investigated whether a relationship between the degree of redness and timing of senescence could be found. Anthocyanins have been suggested to enable a longer growing season by protecting leaves against photo-oxidative stress at the cool temperatures and high irradiance in autumn [17], but for sugar maple a negative association between the time of senescence and contents of anthocyanins have been found [18]. We also investigated whether variation in the amount of anthocyanins in the autumn is associated with leaf traits earlier in the season, and with the amount of anthocyanins in spring leaves.

## 2. Results

### 2.1. Leaf Chlorophyll Content and Timing of Chlorophyll Degradation

**Figure 3:**
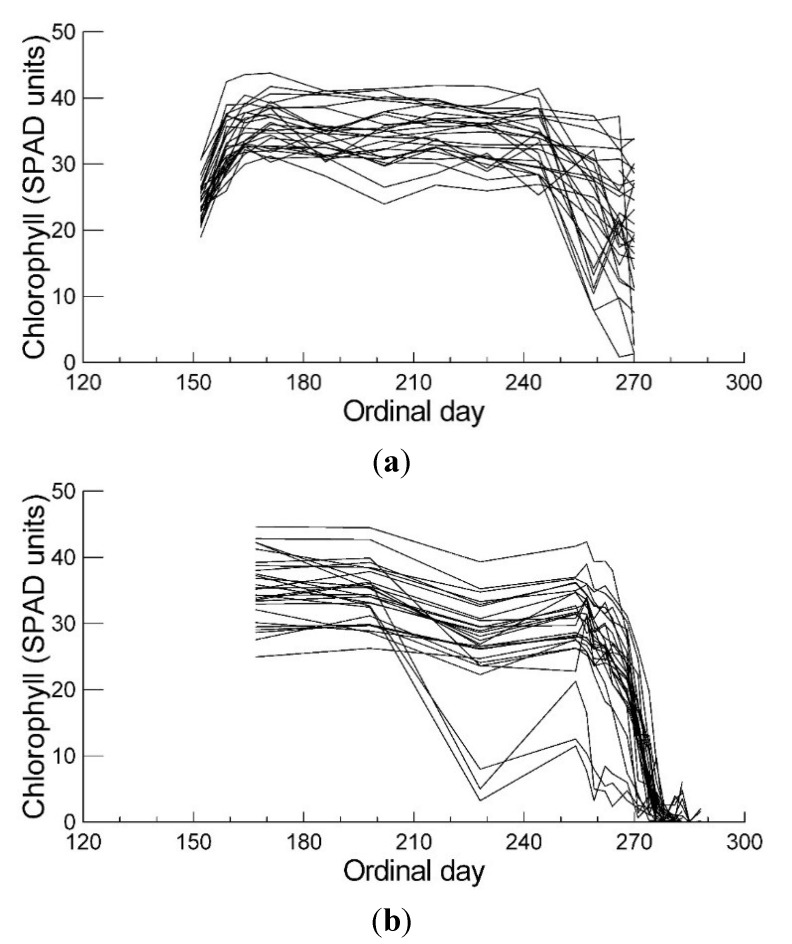
Leaf chlorophyll concentrations in the 27 investigated *Acer rubrum* trees during 2005 (**a**) and 2006 (**b**) expressed as SPAD-units.

Leaf chlorophyll contents were stable from early summer until the end of August, with exception of three trees in 2006, which showed an earlier decline of their chlorophyll (Figure 3a,b). In September the chlorophyll concentrations declined in all trees, but with considerable variation in the extent (Appendix Table A1). The coefficient of variation (CV) of leaf chlorophyll content among the trees was during both summers below 15% (exception: August 2006, 32%), and after mid September above 30%, with values in October 2006 above 100%. Chlorophyll summer values showed a strong correlation between the two years (r = 0.829, *p <* 0.001), but with the earliness of senescence only a weak positive correlation was found, leaves with high chlorophyll content in the summer senescing earlier (r = 0.359, *p <* 0.066). Earliness of senescence correlated negatively with soil pH values, trees on acidic sites senescing earlier. Trees surrounded with more open space senesced earlier, but the correlation was weak (Table 1).

**Table 1:** Correlations of the leaf variables chlorophyll degradation by September, anthocyanin content in autumn, summer chlorophyll content, tannin content, SLA, dry matter content and lamina thickness with the measured environmental factors being canopy openness and soil pH (*n =* 27).

Variable	Canopy Cover		Soil pH	
	r	*p*	r	*p*
Chlorophyll degradation	0.38	0.050 *	−0.54	0.004 **
Anthocyanin	0.44	0.021 *	−0.46	0.017 *
Summer chlorophyll	0.41	0.036 *	−0.40	0.038 *
Tannins	0.22	0.268	−0.40	0.039 *
SLA	−0.83	<0.001 ***	0.34	0.087
DMC	0.54	0.003 **	−0.51	0.007 **
Lamina thickness	0.66	<0.001 ***	0.02	0.907
Chlorophyll degradation	0.38	0.050 *	−0.54	0.004 **

*** *p* < 0.001, ** *p* < 0.01, * *p* < 0.05.

### 2.2. Leaf Anthocyanin Content

**Figure 4:**
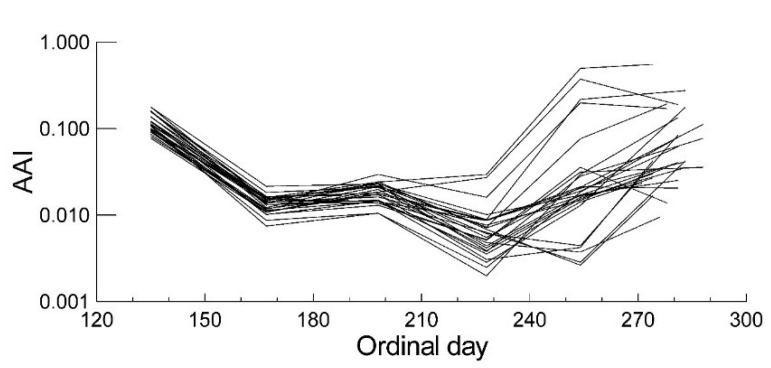
Leaf anthocyanin concentrations in the 27 investigated *Acer rubrum* trees during 2006 expressed as Anthocyanin Absorbance Index (AAI, absorbance at 532 nm minus 24% of absorbance at 653 nm [24]).

There was significant variation among the trees with respect to anthocyanin concentration of the young leaves in spring 2006, and in the autumn in both years, according to ANOVAs with the anthocyanin values as dependent variable and the individual tree identity as dependent factor (spring 2006: r^2^ = 0.914, *p <* 0.001, *n =* 127; ANOVA; autumn 2005: *n =* 123, r^2^ = 0.789, *p <* 0.001; autumn 2006: *n =* 135, r^2^ = 0.942; *p <* 0.001; ANOVA). In 2006, when the full annual course of leaf anthocyanin contents was measured, values decreased from May to June. Variation among the trees remained relatively low until July (Figure 4) with CVs of 25% or below, but in August some trees had already increased their leaf anthocyanins (CV 85%). In September and October an increase in anthocyanins compared to summer values could be observed in all trees, but with a large variation in the extent among the trees, with CVs between 120% and 188%. For 19 out of the 27 trees, spring anthocyanin values were higher than autumn values. Tree averages of autumn anthocyanin contents correlated strongly between the two years (r = 0.854, *p <* 0.001), but there was no significant correlation between the spring and autumn anthocyanin values among the trees, only a weak negative trend (Table 2).

**Table 2:** Correlations of leaf autumn anthocyanin content and earliness of senescence (chlorophyll degradation by mid-late September) with tree height, leaf anthocyanin content in spring, and the summer leaf traits leaf chlorophyll contents, tannin contents, Specific Leaf Area (SLA), dry matter content (DMC), and lamina thickness (*n =* 27).

	Anthocyanins		Senescence	
	r	*p*	r	*p*
Tree height	0.131	0.515	0.361	0.064
Anthocyanins in spring	−0.367	0.060	0.147	0.465
Chlorophyll	0.469	0.014 *	0.254	0.200
Tannins	0.360	0.065	0.274	0.167
SLA	−0.439	0.022 *	−0.354	0.070
DMC	0.419	0.030 *	0.309	0.116
Lamina	0.294	0.137	0.303	0.124

* *p* < 0.05.

Autumn anthocyanin content correlated positively with canopy openness and with soil pH (Table 1), each of the environmental variables explaining about 20% of the variation in individual correlations. A multiple correlation model with both canopy openness and pH explained 31% of anthocyanin variation. The relationships were similar in both years. Spring values of anthocyanin did not correlate with either canopy openness or with soil pH (*p* > 0.75).

**Figure 5:**
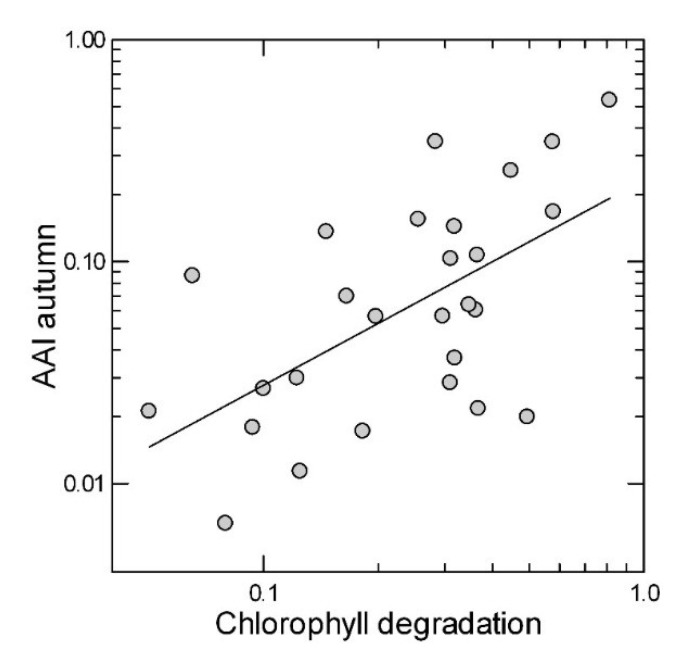
Leaf anthocyanin contents in autumn, expressed as Anthocyanin Absorbance Index (AAI, absorbance at 532 nm minus 24% of absorbance at 653 nm [24]) plotted against earliness of senescence, expressed as the proportion of chlorophyll degraded by mid-September, compared to chlorophyll summer values, among 27 red maple trees. Average values for 2005 and 2006. r = 0.603, *p <* 0.001.

Autumn anthocyanin content had a highly significant correlation with time of senescence, early senescing leaves having higher maximal anthocyanin concentrations than late senescing leaves (Figure 5; Table 2). This relationship was found in both years, but it was stronger in 2006 (r = 0.720, *p <* 0.001) than in 2005 (r = 0.438, *p* = 0.022), possibly due to a longer period of data collection in fall 2006.

### 2.3. Leaf Traits in the Summer

Leaf autumn anthocyanin levels did not correlate with those in the spring, but they did correlate with several leaf traits measured in the summer. Autumn anthocyanin content correlated positively with summer chlorophyll content and leaf dry matter content, and negatively with SLA (Table 2). Leaf tannin contents varied by a factor of 3 (Table A1), and there was a weak positive trend between contents of anthocyanins and tannins (Table 2). All leaf traits correlated with openness of canopy and soil pH, except lamina thickness did not correlate with pH, and tannin contents did not with canopy openness. Time of chlorophyll degradation, *i.e.*, senescence, did not correlate significantly with any of the leaf summer traits (Table 2). Tree height did not correlate with leaf anthocyanin contents, but there was a weak non-significant trend for taller trees to senesce earlier (Table 2). Values for SLA, DMC, lamina thickness and tannins all correlated significantly between the two years (*p <* 0.001).

## 3. Discussion

Our data show a strong link between time of senescence and maximal anthocyanin accumulation in senescing leaves among the studied red maple trees, with early senescing leaves having higher anthocyanin concentrations than late senescing ones. A pronounced development of red colour for early senescing leaves has previously been found for *Acer saccharum* [18] and *Sorbus aucuparia* [25], and was explained as a result of stresses such as N deficiency and cool daytime temperatures, respectively, causing both early senescence and enhanced anthocyanin production. Environmental effects could also be an explanation for the relationship in the present dataset, as both anthocyanin content and time of senescence showed a weak correlation with canopy openness and soil pH. However, as the correlation between anthocyanins and the time of senescence was much stronger than correlations of each of these variables with the environmental variables, we propose a direct functional relationship between the time of senescence and anthocyanin buildup, caused by the stronger solar irradiance earlier in the season.

When photosynthetic machinery is being dismantled, imbalances between energy capture and energy processing lead to increased photo-oxidative stress [26]. Without any control, this would quickly cause senescence to proceed to its terminal stage and its primary function, remobilization of nutrients, would not be fulfilled [13]. Degradation of chlorophyll to colourless breakdown products is an important step to reduce energy capture and buildup of ROS during senescence [27], and the synthesis of anthocyanins is often seen as a further means to reduce the potential photo-oxidative damage, as anthocyanins can screen excessive light and can also act as antioxidants [7]. These two processes are genetically independent [28]. The role of anthocyanins as photo-protective agents seems to be, in at least some cases, senescence-specific, since in *Populus tremula* photo-oxidative stress triggers the buildup of anthocyanins only after a certain date as senescence proceeds [29]. The role of anthocyanins during the autumn senescence is commonly understood to extend the time available to allow efficient nutrient resorption. Nutrient concentrations in senescent leaves have been shown to correlate negatively with anthocyanin content [17] and the abscission layer develops later in red-senescing leaves compared to yellow-senescing leaves when leaves are being compared within trees [30]. Early senescing leaves have been found to have less thorough nutrient resorption than late-senescing leaves in predominantly yellow-senescing species [31].

The earlier senescence of red maple trees with the highest anthocyanin contents as found in the present study, however, indicates that instead of prolonging the growing season for the tree, the role of anthocyanins may rather be protecting plants that for some reason terminate their growing season early. We propose that the stronger solar irradiation in early autumn increases the need to protect early senescing leaves, contributing to the observed variation in the autumn colour among individual trees of red maple. Variation in the time of senescence may have genetic [32] or environmental reasons [18], and may also be a result of the history and general condition of each individual tree, e.g., investment in reproduction [32].

**Figure A1:**
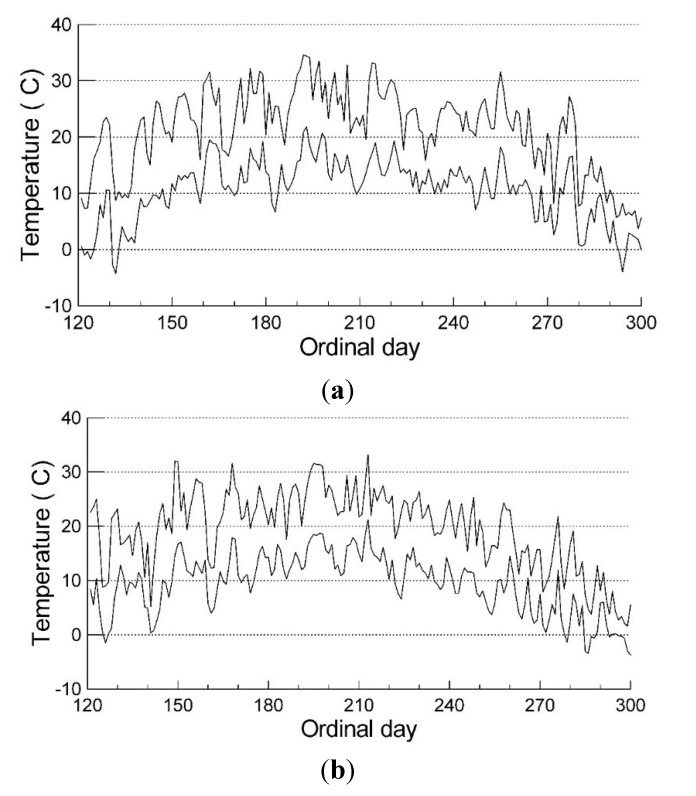
Daily minimum and maximum temperatures during the growing seasons 2005 (**a**) and 2006 (**b**) at the Sudbury airport, 25 km NE from the study site [53].

Photo-protection of senescence is important when there is a high probability of low temperatures coinciding with strong solar radiation, and cool sunny days are known to result in bright fall colours [8]. Such conditions are likely to occur at high elevations [25] and in the deciduous biomes in eastern North America and eastern Asia, especially along their northern parts, which have considerably more southern latitudes than corresponding regions in Europe [33], *i.e.*, cool autumn temperatures coincide with a higher position of the sun. It probably is not a coincidence that these regions are also known for their numerous red-senescing species [6]. During the months of September and October the amount and intensity of solar irradiance diminishes as days become shorter and the position of the sun in the sky gets lower. For example in Sudbury, at the latitude of 46°37’N, day length between 15 September to 15 October is reduced by 13%, but probably more importantly, the intensity of solar irradiance diminishes by 21% due to the lower angle and by 9% due to the longer path through the atmosphere (calculations based on equations in [34]). Due to the larger scatter of blue and green light compared to red [33], this reduction is even stronger for wavelengths absorbed by anthocyanins [35]. Additionally, due to increasing cloud cover the likelihood of bright sunshine diminishes over the course of autumn, e.g., in Sudbury by 17% from September to October [36]. All these factors together make a considerable difference in potential photo-oxidative stress experienced by plants in mid-September, compared to mid-October, dates between which most red maple trees senesce in the region. In mid-August, when the first of our trees increased their anthocyanin contents in 2006, solar radiation is even stronger. The difference between the two years may be a result of slightly cooler temperatures in late summer 2006 (Appendix Figure A1).

The measured environmental variables soil pH and canopy openness had weak effects on both the time of senescence and the levels of anthocyanins. Schaberg *et al.* [18] found similar variation among localities with different N limitation, and Rolshausen and Schaefer [25] among localities with different daytime temperatures. Nitrogen-limitation is often associated with a buildup of anthocyanins [28,37], but in the present study N limitation seems unlikely as a driving force for early senescence and high anthocyanin contents, as anthocyanin levels in the autumn correlated positively with leaf chlorophyll content in the summer. Correlations of these variables with soil pH, however, may be an indication of variation in P limitation. P availability decreases at low pH [38] and low P availability is often associated with dark green leaves and high anthocyanin in general [19,39]. However, as red maple in the region senesces predominantly red, the question with respect to reasons of its colour variation is rather about factors contributing to late senescence and low anthocyanins.

Correlation of anthocyanin contents with canopy openness and with the light-sensitive leaf traits SLA and leaf dry matter content indicates a contribution of the variation in canopy cover to variation in anthocyanin content of the lower canopy leaves. However, as the study trees expressed their different degrees of redness throughout their canopies up to the exposed top, this is unlikely the main reason for the observed variation. The correlation between anthocyanins and tannins was very weak, which was unexpected, as production of these constituents share large parts of their biosynthetic pathways [40], and they have been described to occur often in tandem [41].

Many deciduous trees produce anthocyanins in their leaves not only in the autumn, but also in the young leaves in spring [13]. High anthocyanin levels in spring are associated with photo-protection of leaves before the photosynthetic machinery is fully developed, and interspecific variation (including *A. rubrum*) in the rate of loss of anthocyanins when the season proceeds is closely associated with the rate of leaf maturation [42]. In our dataset, variation in anthocyanin levels in spring was generally lower than in autumn, and the lack of correlation between the spring and autumn anthocyanin values supports earlier findings that this variation in anthocyanin production at different seasons underlies different constraints [43,44].

One factor contributing to the divergent opinions about the functions of anthocyanins in plants is that studies are being compared which have been conducted with different reference systems, *i.e.*, with different species and developmental stages of the plants, and with different stressors [45]. Our dataset from a region with frequent co-occurrence of cool temperatures and strong solar radiation during the senescence supports the idea of a photo-protective role for anthocyanins. This suggests that anthocyanins in red maple are especially important for protection when nutrient remobilization happens while solar radiation is still strong. High levels of anthocyanins in early senescing trees have previously been observed for sugar maple in climatically similar Vermont [18]. Different constraints apply for different climates, e.g., in more southern forests with milder winters, such as North Carolina or Southwest China, anthocyanin buildup in broadleaf evergreen herbs and trees is not limited to the short period of senescence, but reduces the necessity of fall senescence altogether, allowing the plants to maintain functional leaves during the cool season [46,47,48]. On the other hand, many species in northern Ontario senesce yellow, such as silver maple (*Acer saccharinum*) and Manitoba maple (*A. negundo*), the former closely related to *A. rubrum* [21]. This may be due to their somewhat more southern main distribution, but also due to their different ecology. Both species are characteristic of riparian forests, which have been found to have more yellow-senescing species while upland forests rather have red-senescing species [49].

## 4. Experimental Section

### 4.1. Plant Material and Measured Variables

Leaf samples were collected from 27 red maple (*Acer rubrum* L.) trees that ranged from 1 to 12 m in height on the Laurentian University campus in Sudbury, Ontario. Twenty of these trees were within an area of 100 m in diameter (46°28′02″ N, 80° 58′25″ W), and the remaining seven trees 300 m further. The trees were selected in the autumn of 2004 to represent a range of colours from red to yellow. Leaves were collected through the growing seasons 2005 and 2006 from lower branches at 1–2 m height with the highest exposure to sunlight for each individual tree.

Leaf morphological traits were measured excluding the petiole. Leaf dry matter content was measured as leaf dry mass per fresh mass according to the protocol of [50], the Specific Leaf Area (SLA) as leaf area per leaf dry mass (LI-3100 area meter; LI-COR Biosciences, Lincoln, NE, USA) and lamina thickness with a digital micrometer (Mitutoyo Corp., Kawasaki, Japan) as an average of the lateral areas on both sides of a leaf adjacent to the main vein.

Anthocyanins were extracted using the method described in [47] from punched leaf discs of 0.28 cm^2^, and analysed with the protocol described in [24] by measuring the absorbance of the extract at 532 nm and 653 nm (SPECTRO 23 spectrophotometer; Labomed, Inc. Los Angeles, CA, USA). Leaf anthocyanin content is expressed as an Anthocyanin Absorbance Index (AAI), absorbance at 532 nm with a deduction of 24% of the absorbance at 653 nm. Leaf chlorophyll content was measured with a SPAD-502 chlorophyll meter (Minolta Camera Co. Osaka, Japan) as the arithmetic mean of four readings, one from each quadrant of the leaf. Effect of leaf anthocyanin content on the accuracy of chlorophyll determination with SPAD-502 was tested by extracting chlorophyll with the method described in [51] from 30 leaves with varying degrees of redness, and investigating the effect of anthocyanin concentration in the leaf on the relationship between SPAD-values and the values of the extracted chlorophyll. Adding anthocyanin content in a linear regression model between leaf chlorophyll concentration and SPAD readings had no effect on the variation explained by the model (r^2^ = 0.955) and the effect of anthocyanin was non-significant (*p =* 0.877). Tannins were quantified with the method described in [52].

Tree height was measured with a clinometer (Haga, Nürnberg, Germany) on 9 May, 2005. Soil pH at the base of the trees was measured on 16 August 2006 as an average of pH values of soils collected at 2 cm and 10 cm depth on a 1:1 soil:distilled water suspension. Calculation of sunlight exposure of each tree was based on percentage canopy cover, measured using a Model C spherical densiometer (Robert E. Lemmon, Forest Densiometers, Bartlesville, OK, USA) on 16 August 2006, and expressed as canopy openness, calculated as 100% – canopy cover.

### 4.2. Leaf Collection

To assess leaf morphological traits and chlorophyll content, leaves were collected periodically over the 2005 and 2006 growing seasons. In 2005, leaves were collected every two weeks, starting at the beginning of June and continuing until the end of September, the last three harvests weekly. In 2006 the collections were done monthly from mid-June to mid-September. In 2005 three replicate leaves were collected on each occasion, and in 2006 there were ten replicates per collection. Additionally, in 2006 leaf chlorophyll content was measured for 10 leaves on the tree every 2–3 days from 11 September to 15 October, as long as the tree had leaves, the number of replicates being lower if less than ten accessible leaves were left on the tree.

Leaf anthocyanin content was measured on ten replicate leaves collected on 23 August and 5 October in 2005, and with the regular monthly harvests in 2006. In 2006, an additional analysis was conducted on leaves 1–2 weeks after the last regular harvest, depending on the time of abscission. To measure leaf tannin contents, three leaves were collected from each tree in 2005 on 5 July, 5 August, and 5 October, in 2006 with the regular monthly harvests. Leaves used for anthocyanin and tannin quantification were frozen individually in re-closable plastic bags at −20 °C until the analysis within a few months.

### 4.3. Statistical Analyses

For all variables but spring anthocyanin content, which was measured only in 2006, average values of the two years were used. For senescing leaf anthocyanin content, data of the date with the highest average in autumn were used, whereas for all other variables, average values over periods of minimal change during the summer were used. Leaf morphological traits, and tannin and summer chlorophyll contents were averaged over 21 June to 30 August in 2005, and over 12 June to 11 September in 2006. An exception was summer chlorophyll content in 2006 for which June and July values were averaged as some trees already showed a marked decline in August. For autumn chlorophyll the average from 14 September to 28 was calculated in 2005, from 11 September to 28 in 2006. Earliness of senescence was calculated based on the ratio of autumn chlorophyll to summer chlorophyll ratio as 1 − (Chl_autumn_/Chl_summer_). Anthocyanin content, earliness of senescence, canopy openness and SLA values were log transformed to attain normality. All statistical analyses were conducted with SyStat 12.

## 5. Conclusions

The marked variation in autumn colour of red maple (*Acer rubrum*) among individual trees in Sudbury, Ontario, correlates with the time of senescence, with early senescing trees developing higher levels of anthocyanins. A possible factor contributing to this is that early senescing leaves are exposed to higher solar irradiance while dismantling photosynthetic machinery, increasing the danger of photo-oxidative damage.

## Appendix

**Table A1:** The measured traits and environmental variables for the 27 red maple trees: Anthocyanin Absorbtion Index in autumn (AAI Autumn [24]); AAI in May; Proportion of chlorophyll degraded by mid-late September (Senescence; SPAD·SPAD^−1^); Leaf chlorophyll content in summer (Chl.; SPAD units); Tannins (g·g^−1^_Fresh mass_); Specific Leaf Area (SLA; m^2^·g^−1^); Leaf dry matter content (DMC; g·g^−1^); leaf lamina thickness (Lamina; mm), canopy openness (%), soil pH.

Tree #	AAI Fall	AAI May May	Senescence Degradation	Chl. Chlorophyll	Tannins	SLA	DMC	Lamina	Height	Canopy	Soil pH
**1**	0.53	0.12	0.81	38	0.031	12.9	0.45	0.24	9	29	4.4
**2**	0.35	0.09	0.58	32	0.030	14.0	0.45	0.20	9	53	4.2
**3**	0.04	0.10	0.32	39	0.014	13.2	0.44	0.21	10	50	4.7
**4**	0.02	0.10	0.37	30	0.020	19.4	0.39	0.17	7	23	4.8
**5**	0.26	0.09	0.45	40	0.024	13.4	0.44	0.21	7	38	4.8
**6**	0.06	0.12	0.30	42	0.022	12.8	0.46	0.22	8	24	4.8
**7**	0.09	0.08	0.07	36	0.021	16.8	0.41	0.20	1	17	4.7
**8**	0.15	0.09	0.26	36	0.013	26.1	0.37	0.16	5	10	4.7
**9**	0.02	0.08	0.05	35	0.025	18.5	0.42	0.18	8	21	6.5
**10**	0.02	0.12	0.49	31	0.015	21.6	0.37	0.21	12	12	5.1
**11**	0.35	0.11	0.28	37	0.027	18.2	0.40	0.20	4	28	5.3
**12**	0.02	0.16	0.09	34	0.012	22.3	0.38	0.18	11	17	5.1
**13**	0.03	0.11	0.31	39	0.023	17.6	0.41	0.19	4	23	4.6
**14**	0.11	0.09	0.37	39	0.023	15.8	0.41	0.20	5	22	4.6
**15**	0.17	0.14	0.58	36	0.022	17.8	0.45	0.18	3	18	4.3
**16**	0.06	0.10	0.20	34	0.017	20.3	0.40	0.20	8	18	5.1
**17**	0.06	0.12	0.36	34	0.017	20.8	0.40	0.17	2	20	5.1
**18**	0.01	0.14	0.08	26	0.015	26.0	0.37	0.16	1	6	6.0
**19**	0.01	0.16	0.12	29	0.020	22.9	0.39	0.18	1	5	5.9
**20**	0.07	0.09	0.17	31	0.015	20.5	0.39	0.17	3	15	5.3
**21**	0.10	0.16	0.31	35	0.019	14.6	0.41	0.22	4	46	4.6
**22**	0.14	0.11	0.15	36	0.016	16.4	0.41	0.19	6	24	5.4
**23**	0.02	0.18	0.18	30	0.023	15.0	0.42	0.22	2	55	5.3
**24**	0.03	0.18	0.10	32	0.026	14.8	0.43	0.21	2	26	5.0
**25**	0.03	0.16	0.12	40	0.012	14.1	0.42	0.22	4	29	5.0
**26**	0.06	0.10	0.35	33	0.010	16.0	0.38	0.24	7	32	6.3
**27**	0.14	0.11	0.32	35	0.010	14.6	0.39	0.25	7	46	6.0
